# The Cost of Coronavirus Obligations: Respecting the Letter and Spirit of Lockdown Regulations

**DOI:** 10.1017/S096318012000081X

**Published:** 2021-04

**Authors:** DAVID M. SHAW

**Keywords:** coronavirus, pandemic, conflict of interest, ethical analysis

## Abstract

We all now know that the novel coronavirus is anything but a common cold. The pandemic has created many new obligations for all of us, several of which come with serious costs to our quality of life. But in some cases, the guidance and the law are open to a degree of interpretation, leaving us to decide what is the ethical (or unethical but desired) course of action. Because of the high cost of some of the obligations, a conflict of interest can arise between what we want to do and what it is right to do. And so, some people choose to respect only the letter of the law, but not the spirit, or not to respect even the spirit of the guidelines. This paper identifies and describes the new obligations imposed on us all by the pandemic, considers their costs in terms of the good life, and provides an ethical analysis of two personal and two public cases in terms of the letter and spirit of the guidance and legislation.

## Introduction: The Good Life and the New Obligations of Lockdown

Our children attended homeschool this spring. Given the disparity in their ages (9, 6, and 4), philosophy was a great subject to keep them engaged and let them all contribute. In one lesson, they were asked to identify the components of the good life; among others, they came up with parents, home, creating things, having a job, education, family, and friends. But of course, the only reason we were having the lesson was because their school and nursery are closed because of the coronavirus pandemic. And at the end of the class, we went through their list of things they need for a good life again, and highlighted those that have been affected by lockdown. “Playing and having fun” has been curtailed to some extent, as my eldest son cannot play in his football league any more, or go swimming; Cubs and Beavers (Scouts) has also stopped, although the boys still attend virtual meetings; our daughter’s dance classes and planned big show have all been cancelled. “Learning” is still going on, but has clearly been affected substantially (they are certainly more philosophical than they used to be, but I may come to regret teaching them how to argue properly). “Having friends” is also a victim; as Sam said, we still have them, but we cannot see them (according to him, physically distanced playing is not really playing at all). And those are just the ones that are mildly affected; as the kids pointed out, they could not have parties or go to soft play or the cinema; they could not go to nursery or school and see their friends; and they could not even hug their grandparents. Their quality of life lessened somewhat as a result (although it was good to spend so much time together).

Of course, the reason they could not do all these things is because the coronavirus has generated a whole new set of obligations for everyone, which can be summarized by three words: ‘lockdown’ and ‘physical distancing’. Physical distancing is really just one simple obligation: do not go within 2 m of anyone who is not a member of your own household (and the subobligation to wear a facemask or covering if you do need to go closer). From this obligation flow many others, including not hugging your family or friends unless they live with you. And of course, lockdown entails very many more specific obligations, one of which is that you should not be getting anywhere near enough to your family or friends to hug them in any case. When lockdown was introduced, you could only leave the house for a maximum of an hour’s exercise a day. Lockdown also meant that schools and workplaces were closed, which for most families meant that they also had an obligation to juggle working from home with homeschooling, both of which may be entirely new and challenging experiences. Yet another specific and onerous obligation is the duty to self-quarantine and not leave the house for 14 days if you or a family member has any symptoms of the virus.

All of these obligations are very important ones, because if they are not fulfilled the risk of infection rises for both others and one’s family. We clearly have an obligation to help protect public health. But the cumulative cost of all these new obligations is immense, even if self-quarantine remains unnecessary. Not only have we lost many of the key elements of the good life, but also we have so many new positive obligations that stress will inevitably result (even without considering the competing obligations of NHS workers and carers, who must balance personal and family safety against obligations to care for patients^1^, particularly in the absence of personal protective equipment^2^; many patients are also obliged to delay receiving care because nonessential treatment has all but ceased.^3^) Normally, if we were experiencing stress, we could go for a drink with our friends or visit our parents to discuss them; but we cannot, and that is part of the reason for the stress in the first place, one of the paradoxes of the lockdown (another is that working from home would be easy if the grandparents could help with childcare, but of course they cannot because older people are at highest risk). And when people are experiencing stress due to competing obligations, they will try to find ways of resolving the conflicts between these obligations. One way of doing so is to adhere to the spirit, rather than the letter of the law, or to entirely abandon even the spirit of guidelines if a family’s needs are judged to be more important.

## The Letter and the Spirit of Lockdown Legislation and Guidance: Three Cases

When we were in full lockdown, from March until the end of May, the principal instruction was to “stay at home.” This was not only a recommendation, but a legal requirement, according to the Health Protection (Coronavirus) (Restrictions) (Scotland) Regulations 2020: “Except to the extent that a defense would be available under regulation 8(4), F27... no person may leave the place where they are living.”^4^ Regulation 8(4) states that the only exception is when it can be shown that “the person, in the circumstances, had a reasonable excuse,” and provides a list of such excuses, including the need to get food or medical supplies, or to exercise alone or with members of one’s household. Anyone found to be out and about without such a reasonable excuse would be breaking this new law and could be fined.

However, these Regulations were not the only guidance that residents of Scotland were initially expected to obey; they were also told that they should exercise only locally, and not drive to a park or beauty spot: “the Scottish Government advises that you should stay local, use open spaces near to your home where possible and avoid unnecessary travel.”^5^ This meant that we could only use local parks, but the first time we walked to our large local park—the only one suitable for football or cycling—three dogs off the lead distressed our children within the first 10 min. We therefore considered walking up to the meadows, a mile up a steep hill behind our house, but this was potentially a bit too much for our 4-year-old, and the first half of the walk to that park is along busy roads. We therefore thought it justified to drive for 3 min to a street near the park, so that we could all enjoy an hour’s walk in the woods together. This was not against the letter of the law, but it was against the letter of the guidance: we drove to get (nearer) to a local park. However, was it against the *spirit* of the guidance? The instruction not to drive was not written in any document; it was simply conveyed in interviews by the police and some politicians. However, we were told to avoid unnecessary travel. Was it necessary to drive to our local park? Only in the sense that our 4-year-old would not have been able to walk up there, but we were still staying local, and would have walked from the house if she had been able. We did not drive to a nonlocal park, as many did; and if the car had broken down (one of the reasons given for not travelling far, to avoid risk of infecting vehicle rescue workers), we could have walked home down the hill. Is this special pleading?

One of the chief moral charges against those accused of violating guidance (see below) is that they think the rules do not apply to their special circumstances. But we can also assess what we did in terms of universalizability; if it would increase the risk to the public if everyone did what we did, we would clearly be in the wrong. Essentially, we drove for less than 5 min to a park that four out of five family members could have walked to, because one child could not. This enabled us all to have an hour’s walk in the woods, get in touch with nature, and feel refreshed. What if everyone did this? If they only drove for 5 min to get a small child to a local park, would the roads and parks become too busy? Almost certainly not. Thus, our (potential) bending of the rules would not increase the risk of community infection if universalized. Whether or not we were breaking the spirit of the guidelines, we felt guilty and were relieved when, in phase 1 of the exit from lockdown, guidance changed and we could drive up to 5 miles for leisure.

Another personal narrative illustrates the issues in interpreting the letter and spirit of the guidance in phase 1. Some acquaintances drove their children 15 miles to a beach when the guidance was to stay relatively local and not drive more than 5 miles. On the face of it, this is clearly against the letter and the spirit of the guidance; it was not local, and the distance travelled was three times the dictated maximum. In this case, the 5-mile limit was stated on written guidance,^6^ making it a clearer stipulation than the requirement not to drive at all before phase 1 began. However, there was a particular reason for this trip: it was their child’s birthday and they wanted to make it special. Of course, they could have made it special in a different way without travelling so far, but was it against the spirit of the guidance? There is a complicating factor: although 5 miles is the limit for leisure, people in Scotland can travel an unlimited distance to visit family members. In this case, there were no family members on the beach, but the longer excursion was for a family occasion. Thus, although it may seem against the spirit of the leisure guidance, it may be in line with the spirit of the family guidance; indeed, if grandparent from a different household had attended, it could be argued that it would have been entirely within the spirit and letter of the guidance (it does not stipulate in the exception for travel to see family members that it must take place in one of the two dwellings).

This justification certainly seems more like special pleading than in the first case; but what about the universalizability test? It would clearly be bad if everyone felt justified in driving 15 miles to beauty spots; preventing crowds gathering at such locations is the main justification for the limit in the first place (see next section). But if everyone did so because of a child’s birthday, the effect would probably be negligible; and again, if they did so to meet family members (e.g., to minimize distance travelled by one household), it would be within the letter and the spirit of the guidelines even if the effects were more substantial. Nonetheless, by driving so far, our friends did put themselves and their children at slightly higher risk, and they could not know before arriving how busy the beach might be (they travelled after the next case had occurred, though their destination was less popular).

The first weekend of phase 1 in Scotland made it very clear that many people were not applying any universalizability test. Thousands of people drove much more than 5 miles to Loch Lomond and the Trossachs, blocking roads with cars, flooding villages with potentially infected incomers, and leaving behind piles of rubbish and worse in people’s gardens.^7^ Police set up a roadblock in the village of Drymen, turning back anyone who did not live locally; this was not permitted under coronavirus regulations, but justified in virtue of existing traffic laws (due to risk of congestion).^8^ The longer-term result is that the road from Drymen up the eastern side of Loch Lomond was closed for 3 weeks to all but residents.^9^ These members of the public clearly did not care about the letter or the spirit of the guidance; they wanted to have a good time after months of lockdown and the weather was fantastic (and of course, some of them might have had children whose birthday it was). This may be understandable in psychological terms, however unfortunate the consequences. It is also potentially partially explicable by the fourth case we will now discuss, which was so omnipresent in the news in the weeks preceding the beginning of phase 1 that almost all of those breaking the rules at the edge of the Highlands must have been aware of it.

## The Letter and Spirit of Guidance in the Cummings Case: Defending the Indefensible?

Dominic Cummings is the Prime Minister’s Special Adviser. At the end of March, a week into the UK’s lockdown, his wife began to display symptoms of coronavirus. Starting to feel unwell himself, Cummings drove his wife and 4-year-old child to his parents’ farm near Durham, a distance of 260 miles, where they stayed in a separate building from his parents and his sister, who also lives on the farm. They then remained in lockdown there for 14 days.

At first glance, this trip seems entirely contrary to both the spirit and the letter of the lockdown rules. The key message in late March and April was “stay at home,” and that applied to everyone but particularly to those with symptoms of coronavirus and their families. Indeed, the specific guidance was that anyone with symptoms of the virus must self-isolate at home for 7 days without leaving for any reason, and that any family members of those with symptoms must do the same, but for 14 days to allow for any incubation period. No travelling to second homes was permitted for anyone, and particularly not for anyone with symptoms or their families (it has since emerged that Cummings is a co-owner of the property he moved his family to).

Yet Cummings claims and maintains that he “did the right thing.” Why? Because (he claims) he feared that he and his wife were both about to be incapacitated with coronavirus, and would thus be unable to look after their child, and they did not have any friends or family in London who could look after him instead. Is this a reasonable explanation for not staying at home, and driving over 250 miles across England to give the child into the keeping of other people? The explanation will have to be quite robust, as the trip may have put the public at risk. Cummings claims that he did not stop for petrol going in either direction but if he did, he or his family may have passed the virus on to others. Even if they did not stop, passing a child from a family with two symptomatic members to another family would put everyone in that second family at risk—and Cumming’s sister has children too, even if his parents (both over 70 and thus at higher risk) had no contact with any of the London family. Cummings also may have returned to work when he had the virus, and certainly did so after he became sick. Furthermore, there was no clear risk to life as required by the health protection regulations, and social care would have been available in London (where Cumming’s wife’s sister lives). Furthermore, we now know that Cummings also drove 30 miles to Barnard Castle—a nonlocal beauty spot—with his child in the car on his wife’s birthday. His justification for this was that he wanted to test whether his eyesight was good enough to drive back to London for work following his self-isolation. Even if this explanation passed muster, it did not adhere to the letter of the guidance and amounts to special pleading regarding whether it met the spirit (but of course, Cummings is a key worker, and special is even in his job title). An independent fact-checking organization found that “The guidelines accepted that parents in exceptional circumstances would not be able to follow all the rules, but it is not clear whether this applied to Mr. Cummings. Nor did he fully explore the alternatives that might have allowed him to stay in London, which the rules required.”^10^

When news of the Cummings case first broke, it was widely assumed that he would have to resign; the Chief Medical Officer in Scotland had had to do so for visiting a holiday home, and Prof Niall Ferguson, a prominent member of the governmental advisory committee, had also had to resign because his lover travelled to his house with symptoms.^11^ But instead, the government defended Cummings. First, many Cabinet ministers tweeted in support of him, with one saying “Caring for your wife and child is not a crime.”^12^ Instead of any concession that Cummings might have done something wrong, the message was that anyone who thinks he has done something wrong is morally inferior; a cunning inversion. Indeed, the claim became that Cummings was not violating the obligation not to travel; he was fulfilling the obligation to protect a child.

The problem with this narrative is that for over 2 months at this point, everyone in Britain had been being told that many forms of caring for family members were a crime. We were obliged by law not to visit them at all, even if they were dying in hospital; we could not go to their funerals. If we did as Gove suggested, we would all be breaking lockdown rules because we have a greater obligation to our family members. Given that we have not been breaking lockdown rules, the government’s logic is that that must be because we do not love them—an appalling message both in terms of public health compliance and condescension. Not only is the average British citizen wrong to think that Cummings did anything questionable; she was also apparently wrong to avoid missing a loved one’s last breath.

Despite the initial expressions of support from some Conservative ministers and MPs, the growing storm around the Cummings case led many to believe that Johnson would still have to sack him. But in fact, Johnson gave Cummings his wholehearted support, claiming that Cummings had acted “legally and responsibly and with integrity,”^13^ or in other words that he acted both within the letter and the spirit of the law in a way that fulfilled his obligations. But all three of Johnson’s claims are questionable at best. Cummings certainly did not respect the spirit of the guidelines or law, and almost certainly broke them; he did not act responsibly, because in his case that would have meant staying at home and seeking help from family members or social services if his and his wife’s condition worsened considerably. And he did not act with integrity, because he acted illegally and irresponsibly, without due consideration of his obligations.

Johnson also claimed that his adviser had “acted by instinct as any father would.” This is problematic for at least three reasons. First, it is extremely offensive to fathers and others who have spent 2 months suppressing their instincts about seeing family members in order to obey the very guidelines that Johnson and Cummings designed. Second, if the Prime Minister is endorsing acting out of concern for family members when faced with a decision about the virus, that will severely weaken compliance with lockdown restrictions; why should people obey the instruction to avoid meeting family members in large groups if doing so feels right? More generally, why obey the rules when the Prime Minister is making excuses for a rule breaker? As events near Loch Lomond show, these are not theoretical questions.

And third, the very last message that a political leader should be sending in a public health emergency is that acting on instinct is responsible. It is not; it is irresponsible. Acting with integrity, as the PM claims Cummings did, means acting reflectively, not intuitively, and considering our competing obligations in light of all the rules and risks. Instead, he acted by instinct and increased the risk to his child and others. This appalling public health messaging is made even worse by the Prime Minister’s recent claim that “what is needed is good solid British common sense.”^14^ Apparently, that means ignoring lockdown obligations, putting your child at increased risk by putting him in a car with an infected person, and driving halfway across the country to possibly infect other members of the public as well as other family members. Johnson had a clear obligation to sack Cummings, but instead he gave into his conflict of interest and did what was worse for public health—the very opposite of what the British public are expected to do. In defending someone who entirely failed the universalizability test, Johnson effectively legitimized universal bad behavior. Table 1 summarizes the four cases and Johnson’s actions, whether the spirit and letter of the law and guidance was respected in each, and whether there was any risk to public health as a result.

**Table 1.** Respecting the letter and spirit of regulations in the four cases.

	Letter of the law	Spirit of the law	Risk to public health
Driving to get to local park	No	Yes	No
Driving beyond 5-mile limit	No	Partially	Very low
Flocking to Loch Lomond	No	No	High
Driving to Durham & Barnard Castle eye test	No (but claimed yes)	No	Medium (but led to e.g., Loch Lomond)
Defending driving to Durham and Barnard Castle	No	No	Extremely high

## Conclusion

One element of the good life that my children did not immediately identify was helping people. The vast majority of people in Britain and across the globe are doing their best to do just that by fulfilling all the new obligations imposed upon them by the coronavirus pandemic in order to help protect public health, whatever the cost to their quality of life. Some will bend the guidelines a little to fulfil other obligations. But it is imperative for those who set our new imperatives to obey them, or we run the risk of having very few people respect either the letter or the spirit of the new rules that are designed to save lives and someday enable a return to normal life.

## Notes

1. McConnell D. Balancing the duty to treat with the duty to family in the context of the COVID-19 pandemic. Journal of Medical Ethics 2020;46:360–3.3233215410.1136/medethics-2020-106250PMC7211094

2. Schuklenk U. What healthcare professionals owe us: Why their duty to treat during a pandemic is contingent on personal protective equipment (PPE). Journal of Medical Ethics 2020;46:432–5.3244442510.1136/medethics-2020-106278

3. Shaw D. The vital contexts of coronavirus. *Journal of Medical Ethics* blog; 2nd April 2020; available at https://blogs.bmj.com/medical-ethics/2020/04/02/the-vital-contexts-of-coronavirus/ (last accessed 8 July 2020).

4. Scottish Parliament. *The Health Protection (Coronavirus) (Restrictions) (Scotland) Regulations 2020. Section 5.1*; available at http://www.legislation.gov.uk/ssi/2020/103#commentary-key-5e0a325405e421896a160e11a7c8dace (last accessed 8 July 2020).

5. Police Scotland. *Covid 19 FAQs. Covid-19 and Non-Essential Travel*; available at https://www.scotland.police.uk/about-us/covid-19-policescotlandresponse/faqs-essential-and-non-essential-travel (last accessed 8 July 2020).

6. Scottish Government. *Coronavirus (COVID-19): Framework for Decision Making – Scotland’s Route Map Through and Out of the Crisis*; available at https://www.gov.scot/publications/coronavirus-covid-19-framework-decision-making-scotlands-route-map-through-out-crisis/pages/7/ (last accessed 8 July 2020).

7. Cassidy R. Shocking pictures show rubbish carnage around Scots beauty spots as lockdown restrictions ease. *Daily Record*; 3 June 2020; available at https://www.dailyrecord.co.uk/news/scottish-news/shocking-pictures-show-rubbish-carnage-22130881 (last accessed 8 July 2020).

8. Carrell S. Travel in Scotland may be restricted after hundreds flout rules. *The Guardian*; 1 June 2020; available at https://www.theguardian.com/uk-news/2020/jun/01/travel-in-scotland-may-be-restricted-after-hundreds-flout-lockdown-rules (last accessed 8 July 2020).

9. British Broadcasting Corporation. Roads closed at Scottish beauty spots as people warned away. *BBC*; 6 June 2020; available at https://www.bbc.co.uk/news/uk-scotland-52948072 (last accessed 8 July 2020).

10. Full Fact (fact-checking website). What did the lockdown rules say when Dominic Cummings travelled to Durham?; 27 May 2020; available at https://fullfact.org/health/dominic-cummings-lockdown-rules/ (last accessed 8 July 2020).

11. Perraudin F. The high-profile figures who got into hot water over lockdown rules. *The Guardian*; 5 May 2020; available at https://www.theguardian.com/world/2020/may/05/the-high-profile-figures-who-got-into-hot-water-over-lockdown-rules (last accessed 8 July 2020).

12. Gove M. *Twitter*; 23 May 2020; available at https://twitter.com/michaelgove/status/1264126108733186050 (last accessed 8 July 2020).

13. Otte J, Walawalkar A. Boris Johnson says Dominic Cummings acted ‘responsibly, legally and with integrity’. *The Guardian*; 24 May 2020; available at https://www.theguardian.com/world/live/2020/may/24/uk-coronavirus-live-dominic-cummings-under-intense-pressure-over-lockdown-breaches.

14. Shaw D. The UK government is encouraging people outside England to break lockdown rules. *Journal of Medical Ethics* blog; 20 May 2020; available at https://blogs.bmj.com/medical-ethics/2020/05/20/3835/ (last accessed 8 July 2020).

